# Activation of 3-Mercaptopyruvate Sulfurtransferase by Glutaredoxin Reducing System

**DOI:** 10.3390/biom10060826

**Published:** 2020-05-28

**Authors:** Noriyuki Nagahara

**Affiliations:** Isotope Research Laboratory, Nippon Medical School, 1-1-5 Sendagi Bunkyo-Ku, Tokyo 113-8602, Japan; noriyuki@nms.ac.jp; Tel.: +81-3-3822-2131

**Keywords:** glutaredoxin, glutathione, glutathione reductase, 3-mercaptopyruvate sulfurtransferase

## Abstract

Glutaredoxin (EC 1.15–1.21) is known as an oxidoreductase that protects cysteine residues within proteins against oxidative stress. Glutaredoxin catalyzes an electron transfer reaction that donates an electron to substrate proteins in the reducing system composed of glutaredoxin, glutathione, glutathione reductase, and nicotinamide-adenine dinucleotide phosphate (reduced form). 3-mercaptopyruvate sulfurtransferase (EC 2.8.1.2) is a cysteine enzyme that catalyzes transsulfuration, and glutaredoxin activates 3-mercaptopyruvate sulfurtransferase in the reducing system. Interestingly, even when glutathione or glutathione reductase was absent, 3-mercaptopyruvate sulfurtransferase activity increased, probably because reduced glutaredoxin was partly present and able to activate 3-mercaptopyruvate sulfurtransferase until depletion. A study using mutant *Escherichia coli* glutaredoxin1 (Cys^14^ is the binding site of glutathione and was replaced with a Ser residue) confirmed these results. Some inconsistency was noted, and glutaredoxin with higher redox potential than either 3-mercaptopyruvate sulfurtransferase or glutathione reduced 3-mercaptopyruvate sulfurtransferase. However, electron-transfer enzymatically proceeded from glutaredoxin to 3-mercaptopyruvate sulfurtransferase.

## 1. Introduction

Glutaredoxin (Grx) was first discovered by Holmgren [1,2,3]. Grx is an enzyme and is categorized as an oxidoreductase (EC 1.15–1.21) that catalyzes not only the reduction process of a disulfide bond on the substrate protein but also cleavage of glutathione (GSH) from the GSH-disulfide complex, meaning that Grx enzymatically donates electrons to substrate proteins; the full reaction mechanism has been clarified [4,5,6,7]. Protein substrates are reduced by Grx, and then oxidized Grx is non-enzymatically reduced by GSH, a redox molecule. Oxidized GSH is enzymatically reduced by glutathione reductase (GRD) together with nicotinamide-adenine dinucleotide phosphate in its reduced form (NADPH) as a coenzyme. Many eukaryotic and prokaryotic subtypes of Grx have been reported; among these subtypes, a ternary structure for *Escherichia coli* Grx1 has been reported [8]. This study clarified that Cys^14^ was not only a binding site for GSH but also the site of a disulfide bond with Cys^11^. These facts were confirmed by a study using mutant *E. coli* Grx1 (Cys^14^ was replaced with Ser) [9]. As a redox protein like Grx, thioredoxin (Trx) is well studied and is a smaller molecule with lower redox potential. Trx also reduces substrate proteins associated with Trx reductase (TRD).

3-mercaptopyruvate sulfurtransferase (MST, EC 2.8.1.2) catalyzes a transsulfuration. Recently, it has been revealed that MST produces hydrogen sulfide and polysulfides [10,11,12,13,14,15,16,17,18] and plays important roles in living organisms [19,20,21,22,23,24,25,26,27,28,29,30,31,32,33,34,35,36,37,38,39,40,41,42,43]. MST is activated by the Trx-TRD, but not the GSH-GRD, reducing system due to differences in redox potential [44,45,46,47]. There are two target points for the reduction in MST; a sulfenic acid formed at the catalytic site cysteine and a disulfide bond in dimeric MST [44,45,46,47]. MST activity is regulated via redox-dependent changes at these molecular points at the post-translational level [44,45,46,47]. In this experiment, we proved that Grx reduced oxidized-MST to activate MST, although the redox potentials of human Grx1 and Grx2 were −232 mV and −222 mV, respectively [48], and that of *E. coli* Grx1 was −233 mV [49], which was higher than those of GSH (~−240 mV) [48] and Trx (~−270 mV) [38].

## 2. Materials and Methods

### 2.1. Overexpression and Preparation of Rat MST

A pET28a vector containing rat MST was introduced in *E. coli*, BL21(DE3) cells transformed with a pSTV vector containing GroEL and GroES cDNAs. MST was purified according to a procedure described previously [44,45]. The resulting MST was 131 μM in 20 mM in potassium phosphate buffer, pH 7.2, and was frozen until use.

### 2.2. Preparation of Other Proteins and Chemical Agents

Human Grx1 (IMCO Corporation Ltd AB, Stockholm, Sweden) and human Grx2 (IMCO Corporation Ltd AB, Stockholm, Sweden) were adjusted to 1 mM each using 20 mM potassium phosphate buffer, pH 7.2, as a stock solution. *E. coli* Grx1 (IMCO Corporation Ltd AB, Stockholm, Sweden) and *E. coli* mutant Grx (C14S, Cys^14^ of *E. coli* Grx1 was replaced with Ser) (IMCO Corporation Ltd AB, Stockholm, Sweden) were adjusted to 3 mM each with 20 mM potassium phosphate buffer, pH 7.2. Human GRD (Sigma-Aldrich, Inc. St. Louis, MO, USA) and *E. coli* GRD (Novus Biologicals, Centennial, CO, USA) were 304 μM and 19.531 μM, respectively, in their original solutions. NADPH (Sigma-Aldrich, Inc. MO, USA) and GSH (Sigma-Aldrich, Inc.) were adjusted to 10 mM each with potassium phosphate buffer, pH 7.2. Sodium thiosulfate, sodium cyanide, and other chemicals were purchased from Wako Pure Chemicals Industries, Ltd. (Osaka, Japan).

### 2.3. Grx-GSH-GRD-NADPH Reducing Systems

In the Grx, GSH, GRD, and NADPH systems, all reagents were further diluted with 20 mM potassium phosphate buffer, pH 7.2. MST was diluted to 1/50-fold molar concentration (final concentration; 2.62 μM), human Grx1 to 1/100-fold molar concentration (final 10 μM), human Grx2 to 100-fold molar concentration (final 10 μM), *E. coli* Grx1 to 1/100-fold molar concentration (final 30 μM), *E. coli* mutant Grx to 1/100-fold molar concentration (final 30 μM), human GRD to 1/100-fold molar concentration (final 3.04 μM), *E. coli* GRD to 1/100-fold molar concentration (final 0.2 μM), and NADPH to 1/100-fold molar concentration (final 100 μM). GSH (10 mM) was used without dilution.

The incubation mixtures for the study of activation contained 1 μL of diluted MST solution, 1.6 μL of diluted NADPH solution, 1.2 μL of diluted human Grx1 (or human Grx2) solution, 0.9 μL of human GRD solution, and 1 μL of diluted GSH solution. Other incubation mixtures contained 1 μL of diluted MST solution, 1.6 μL of diluted NADPH solution, 1 μL of diluted *E. coli* Grx1 (or *E. coli* Grx1 mutant) solution, 1.3 μL of *E. coli* GRD solution, and 1 μL of diluted GSH solution. As control experiments, after the incubation of MST with 1.2 mM dithiothreitol (DTT) for 20 min on ice, DTT was removed on a PD 10 (GE Healthcare, Chicago, IL, USA). Then, MST activity was measured. After treatment of human Grx 1 with 1.2 mM DTT for 20 min on ice, DTT was removed on a PD 10, and Grxs were concentrated with VIVASPIN 500, MWCO 5000, PES (Sartorius AG, Goettingen, Germany). Then, MST was mixed with DTT-pretreated Grxs, and MST activity was measured. The total volume of each incubation mixture was adjusted to 20 μL using 20 mM potassium phosphate buffer, pH 7.2.

### 2.4. Time Dependency of MST Activation

The contents of the incubation mixture were as described above. After incubation at 20 °C for 0, 5, 10, and 15 min, individual 5 μL aliquots were used to measure enzyme activity (rhodanese activity).

### 2.5. MST Activation Studies Using the Reducing System

#### 2.5.1. Enzyme Activation in Human Grx1 System with or without GSH

(a) Measurements of MST activity under various human Grx1 concentrations without GSH: The incubation mixture contained 0, 0.3, 0.6, 0.9, 1.2, or 1.5 μL of human Grx1 solution in 20 μL of incubation mixture (final concentrations: 0, 0.15. 0.3, 0.45, 0.6, and 0.75 μM, respectively) without GSH solution. After incubation at 20 °C for 10 min, 5 μL aliquots were used to measure enzyme activity (rhodanese activity).
(b) Measurements of MST activity under various NADPH concentrations without GSH: The incubation mixture contained 0, 0.4, 0.8, 1.2, 1.6, or 2.0 μL of NADPH solution in 20 μL of incubation mixture (final concentrations: 0, 2, 4, 6, 8, and 10 μM, respectively) without GSH solution.
(c) Measurements of MST activity under various human GRD concentrations without GSH: The incubation mixture contained 0, 0.3, 0.6, 0.9, 1.2, or 1.5 μL of human GRD solution in 20 μL of incubation mixture (final concentrations: 0, 0.0456, 0.0912, 0.1368, 0.1824, and 0.228 μM, respectively) without GSH solution.
(d) Measurements of MST activity under various GSH concentrations: The incubation mixture contained 0, 0.2, 0.5, 1, 1.5 or 2 μL of GSH solution in 20 μL of incubation mixture (final concentrations: 0, 0.1, 0.25, 0.5, 0.75, and 1 mM, respectively).

#### 2.5.2. Enzyme Activation in a Human Grx2 System with or without GSH

The incubation mixture contained 0, 0.3, 0.6, 0.9, 1.2, or 1.5 μL of human Grx2 solution in 20 μL of incubation mixture (final concentrations: 0, 0.15. 0.3, 0.45, 0.6, and 0.75 μM, respectively) with or without 1 μL of GSH solution.

#### 2.5.3. Enzyme Activation in an E. coli Grx1 System with or without GSH

The incubation mixture contained 0, 0.5, 1, 1.5, 2, or 2.5 μL in 20 μL of *E. coli* Grx1 solution and 20 μL of incubation mixture (final concentrations: 0, 0.75, 1.5, 2.25, 3, and 3.75 μM, respectively) with or without 1 μL of GSH solution.

#### 2.5.4. Enzyme Activation in an E. coli Grx1 Mutant System with or without GSH

The incubation mixture contained 0, 0.5, 1, 1.5, 2, or 2.5 μL in 20 μL of *E. coli* Grx1 mutant solution and 20 μL of incubation mixture (final concentrations: 0, 0.75, 1.5, 2.25, 3 and 3.75 μM, respectively) with or without 1 μL of GSH solution.

### 2.6. Rhodanese Activity of MST

In usual MST activity when catalyzing the trans-sulfuration from mercaptopyruvate to 3-mercaptoethanol, 3-mercaptoethanol can reduce agents including MST during incubation in the assay mixture. As MST possesses rhodanese activity [50,51] via catalyzing the trans-sulfuration from thiosulfate to cyanide, the rhodanese activity of MST was measured in this experiment. All activities were measured in triplicate.

### 2.7. Protein Concentration

The protein concentration was determined with a Coomassie protein assay kit (Pierce Biotechnology, Rockford, IL, USA) using crystalline bovine serum albumin (ICN Biochemicals, Irvine, CA, USA) as the standard.

### 2.8. Statistical Analysis

All values are expressed as the mean ± S.D. Significance of difference between values was estimated by a Student’s t-test. A *p*-value of less than 0.05 was considered statistically significant.

## 3. Results and Discussion

### 3.1. Time-Dependent MST Activation

**Figure 1:**
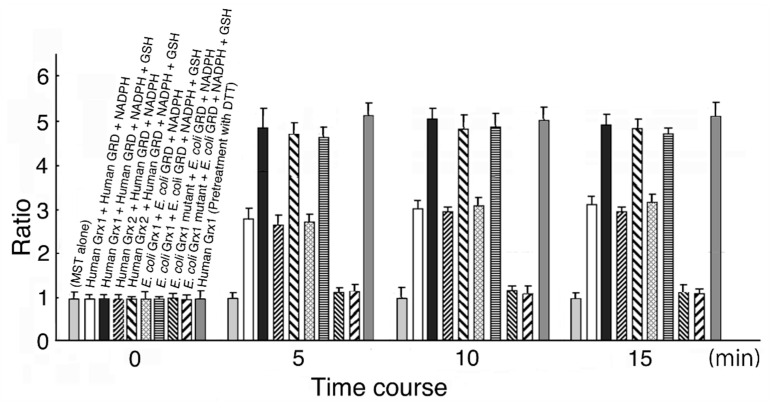
Time-dependent MST activation. The MST reducing system contains Grx, GSH, GRD, and NADPH. Each system was incubated at 20 °C for 0, 5, 10, and 15 min. Details are described in the text.

MST activation was completed at around 5 min, and therefore, we measured activity at 10 min in this experiment. As shown in Figure 1, MST activity was increased by 3.01-fold in the reducing system containing human Grx1 (final concentration 0.6 μM), human GRD (final concentration 90 μM), and NADPH (final concentration 8 μM); by 4.13-fold in the system containing human Grx1, human GRD, NADPH, and GSH (final concentration 0.5 mM); by 2.95-fold in the system containing human Grx2 (final concentration 0.6 μM), human GRD, and NADPH; by 3.92-fold in the system containing Grx2, human GRD, NADPH, and GSH; by 3.02-fold in the system containing *E. coli* Grx1 (final concentration 1.5 μM) (*p* = 1.69 × 10^−1^), *E. coli* GRD, and NADPH; and by 1.10-fold in the system containing *E. coli* Grx1 mutant, *E. coli* GRD, NADPH, and GSH (*p* = 3.52 × 10^−1^). When MST was treated with 1.2 M DTT, MST activation increased to about 1.5-fold of control, consistent with the previous results [44] (data not shown). Further, when human Grx 1 was pretreated with 1.2 mM DTT, MST activity increased to about 5-fold of control, also consistent with the previous results [44]. Even when the reducing system did not contain GSH, MST activity was increased. This is probably because residual Grx in a reduced form donates electrons to MST until depleted. When GSH was present in the system, activity increased to between 1.58- and 1.70-fold of the values without GSH.

In our previous studies [44,45,46,47], MST was reversely inhibited by a stoichiometric concentration of hydrogen peroxide or oxygen. MST is oxidized to form a sulfenate at the catalytic site cysteine. Furthermore, MST was oxidized to form sulfinate and/or sulfonate to be inactivated. Conversely, MST contained a small amount of these pieces under the present experimental conditions. On the other hand, dimer–monomer equilibrium is also involved in MST activity regulation (in overexpressed MST under air-saturated conditions; monomeric MST to dimeric MST = ~10:1 [44]). The observed MST activation may be due to not only the reduction of a sulfenic acid formed at the catalytic site cysteine but also the reduction of a disulfide bond in dimeric MST [45,46,47]. Grxs may contribute to the dimer–monomer equilibrium of MST. Previous studies confirmed that thioredoxin (Trx) also activates MST via not only the reduction of a sulfenic acid formed at the catalytic site cysteine but also the reduction of a disulfide bond in dimeric MST [45,46,47]. Interestingly, eukaryotic MST is much more effectively activated by prokaryotic Trx than by the eukaryotic one [44]. Conversely, eukaryotic MST is more effectively activated by eukaryotic Grx than by the prokaryotic one.

The *E. coli* Grx1 mutant had little effect on MST activity with GSH. MST activity with or without GSH after a 10 min incubation increased to 1.24- or 1.16-fold, respectively from before incubation; however, the difference was not significant (*p* = 9.21 × 10^−2^ and *p* = 8.52 × 10^−2^, respectively). Bushweller et al. [9] reported that Cys^14^ on *E. coli* Grx1 was a binding site for GSH and replacement of the Cys with Ser decreased activity to 38% of full.

Considering the redox potential [45,46,47] of the inactive form, oxidized MST (a low redox potential sulfenate formed at the catalytic site cysteine and/or disulfide bridge in dimeric MST) can be reduced and reactivated through accepting electrons from the Trx-TRD system, but not from the GHS-GRD system, because the redox potential of MST is lower than that of GSH (~−240 mV) [48] and higher than that of Trx (~−270 mV) [48]. The findings of this experiment show that the Grx-GSH-GRD system can also reduce MST, despite Grx possessing a higher redox potential (~−200~−233 mV) [48,49]. This is probably because MST and Grx are cross-compatible in their substrate–enzyme interaction.

### 3.2. Human Grx1 and GSH Dose-Dependent MST Activation

**Figure 2:**
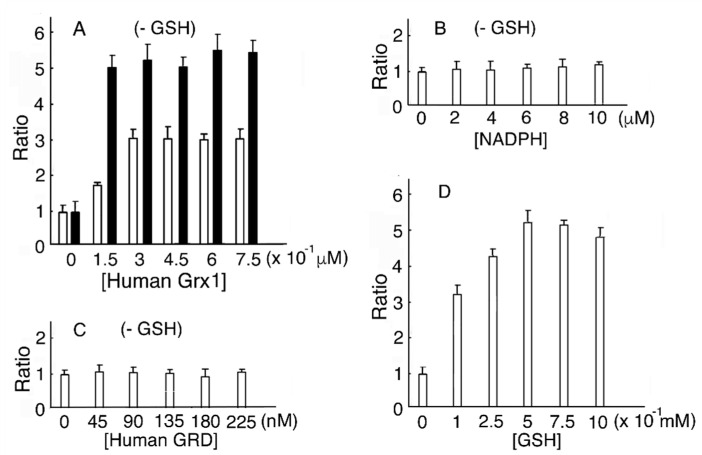
Human Grx1 and GSH dose-dependent MST activation. (**A**). The MST reducing system contains various concentrations of human Grx1, human GRD, and NADPH without GSH. (**B**). The MST reducing system contains human Grx1, human GRD, and various concentrations of NADPH without GSH. (**C**). The MST reducing system contains human Grx1, various concentrations of human GRD, and NADPH without GSH. (**D**). The MST reducing system contains human Grx1, human GRD, and NADPH with various concentrations of GSH. 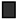 Incubation with DTT-pretreated human Grx1.

In a reducing system containing human Grx1, human GRD, and NADPH without GSH, MST activity reached a maximum value (2.91-fold of that without human Grx1) at 0.45 μM human Grx1. As a control, MST was incubated with DTT-pretreated human Grx1. The ratio of MST activity showed a maximum value (5.2) (Figure 2A). Residual reduced human Grx1 donates electrons to MST until depleted. When the reducing mixture contained human Grx1, human GRD, and NADPH without GSH, various concentrations of NADPH or human GRD induced no peak in MST activity (Figure 2B,C). It is reasonable to conclude that the absence of Grx does not change MST activity. When various concentrations of GSH were present in the mixture, MST activity reached a maximum (5.32-fold of without GSH) at 0.5 mM GSH (Figure 2D). Our results showed that MST activity at 0.45 μM human Grx1 and 0.5 mM GSH increased to 1.83-fold of 0.45 μM human Grx1 without GSH (*p* = 6.15 × 10^−3^).

### 3.3. Human Grx2 and GSH Dose-Dependent MST Activation

**Figure 3:**
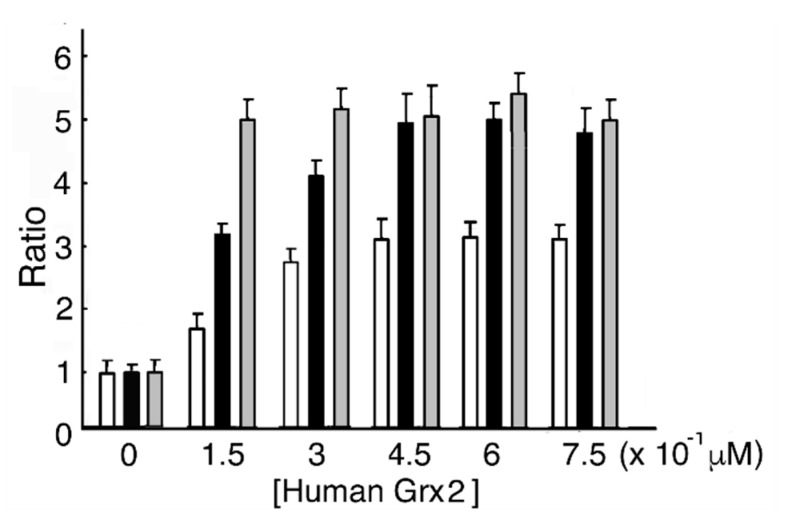
Human Grx2 and GSH dose-dependent MST activation. One MST reducing system contains various concentrations of human Grx2, human GRD, NADPH, and GSH, while the other reducing system contains human Grx2, human GRD, NADPH, and various concentrations of GSH. Each mixture was incubated at 20 °C for 10 min. The mixture is with (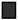) or without (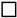) GSH. (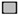), Incubation with DTT-pretreated human Grx2.

In the reducing system containing human Grx2, human GRD, and NADPH with or without GSH, MST activity reached a maximum value (4.93- and 3.06-fold of that without human Grx2, respectively) at 0.45 μM human Grx2. As a control, MST was incubated with DTT-pretreated human Grx2. The ratio of MST activity showed a maximum value (5.5) (Figure 3). Our results show that MST activity at 0.45 μM human Grx2 and 0.5 mM GSH increased to 1.61-fold of 0.45 μM human Grx2 without GSH. (*p* = 9.47 × 10^−3^). When the reducing mixture did not contain GSH, residual reduced human Grx2 donated electrons to MST until depletion. Compared to human Grx1, Grx2 exhibited no difference in function from its counterpart.

### 3.4. *E. coli* Grx1 and GSH Dose-Dependent MST Activation

**Figure 4:**
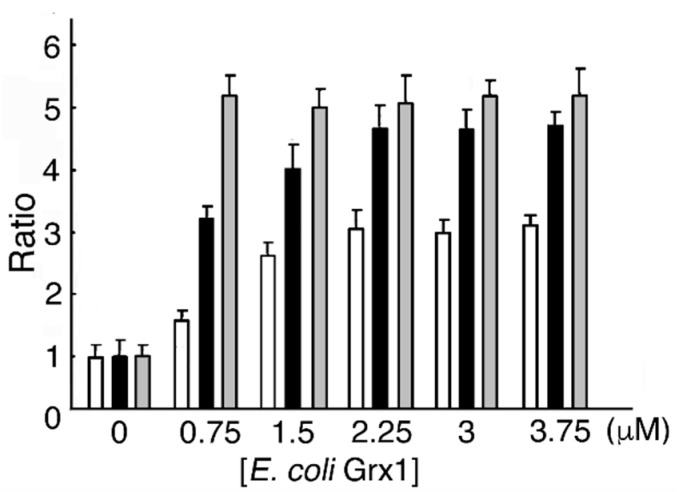
*Escherichia coli* Grx1 and GSH dose-dependent MST activation. One MST reducing system contains various concentrations of *E. coli* Grx1, *E. coli* GRD, NADPH, and GSH, while the other reducing system contains *E. coli* Grx1, *E. coli* GRD, NADPH, and various concentrations of GSH. Each mixture was incubated at 20 °C for 10 min. The mixture is with (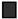) or without (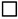) GSH. (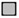) , Incubation with DTT-pretreated *E. coli* Grx1.

In a reducing system containing *E. coli* Grx1, *E. coli* GRD, and NADPH with or without GSH, MST activity reached a maximum value (4.78- and 3.03-fold of that without *E. coli* Grx1, respectively) at 2.25 μM *E. coli* Grx1. As a control, MST was incubated DTT-pretreated *E. coli* Grx1. The ratio of MST activity showed a maximum value (5.4) (Figure 4). Our results show that MST activity at 2.25 μM *E. coli* Grx1 and 0.5 mM GSH increased to 1.56-fold of that at 2.25 μM *E. coli* Grx1 without GSH (*p* = 7.47 × 10^−2^). When the reducing mixture did not contain GSH, residual reduced *E. coli* Grx1 donated electrons to MST until depletion. Compared to eukaryotic Grxs, about 5 times the amount of *E. coli* Grx1 is required to serve as an equivalent electron donor for eukaryotic MST. This experiment suggests that prokaryotic Grx is less effective on eukaryotic proteins than the eukaryotic form. This finding is opposite to the results for prokaryotic Trx, which more effectively reduces eukaryotic proteins than does eukaryotic Trx; this difference is probably due to structural properties [44,45,46,47].

### 3.5. *E. coli* Grx1 Mutant and GSH Dose-Dependent MST Activation

**Figure 5:**
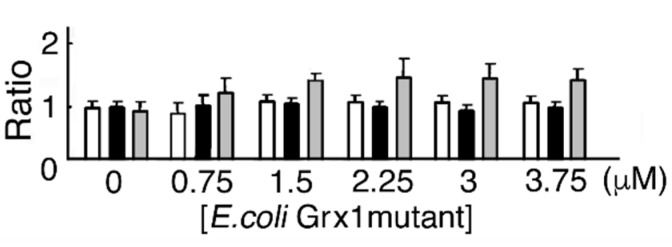
*E. coli* Grx1 mutant and GSH dose-dependent MST activation. One MST reducing system contains various concentrations of *E. coli* Grx1 mutant, *E. coli* GRD, NADPH, and GSH. The other reducing system contains *E. coli* Grx1 mutant, *E. coli* GRD, NADPH, and various concentrations of GSH. Each mixture was incubated at 20 °C for 10 min. The mixture is with (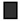) or without (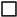) GSH. (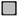), Incubation with DTT-pretreated *E. coli* Grx1 mutant.

In the reducing system containing *E. coli* Grx1 mutant, *E. coli* GRD, and NADPH with or without GSH, MST activity was not affected. As a control, MST was incubated with DTT-pretreated *E. coli* Grx1 mutant. The ratio of MST activity showed a maximum value (1.5) (Figure 5). As discussed above, mutant Grx C14S can maintain 38% of enzyme activity to donate electrons to MST [9], but it hardly works as an electron donor.

## 4. Conclusions

**Figure 6:**
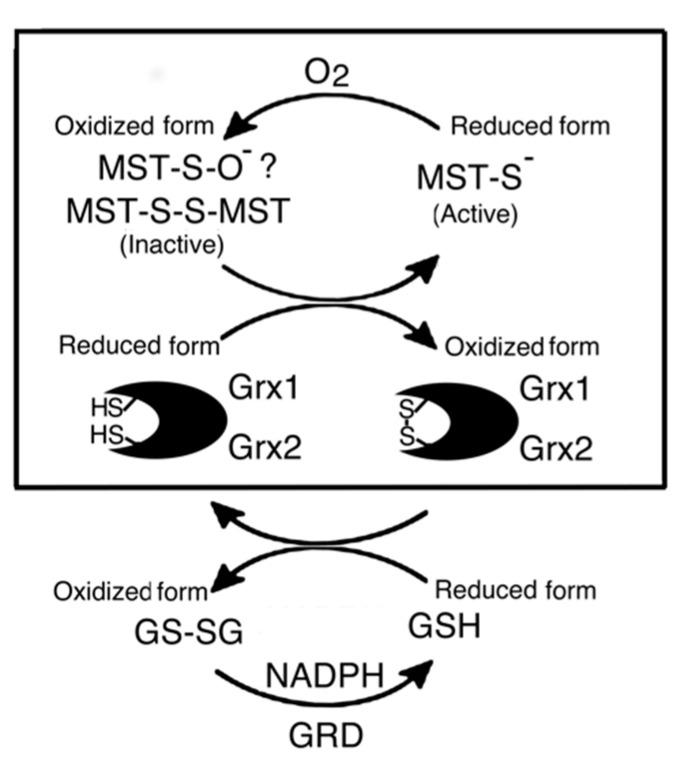
Probable activation mechanism of MST in the Grx-GSH-GRD-NADPH reducing systems. Box: The reducing system contains Grx without Grx, GRD, and NADPH. Details are described in the text.

**Figure 7:**
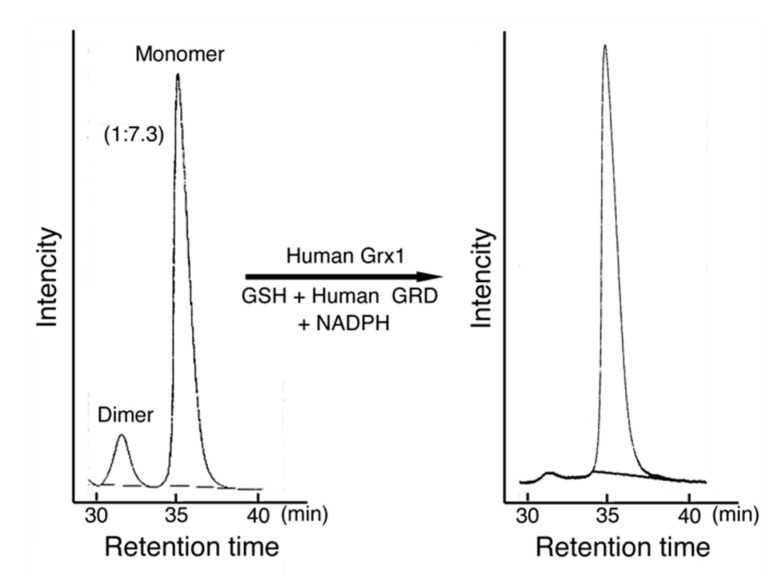
Preliminary study on the activation mechanism of MST. MST was mixed with a human Grx1 mixture containing GSH, human GRD, and NADPH for 20 min on ice. The mixture and control MST were analyzed using an HPLC system (Hitachi Chromaster system, Tokyo, Japan) with two TSK gel filtration columns connected in series (G3000SW_xL_, 7.8 mm × 30 cm and G2000SW_xL_, 7.8 mm × 30 cm, TOSOH Corp., Tokyo, Japan). The mobile phase was 0.2 M potassium phosphate buffer, pH 7.2. The flow rate was 0.5 mL/min. The detection was made at 280 nm. In this case, MST contained 13.6% of the dimer. The dimer MST was reduced to monomer MST and MST activity was increased.

MST is activated in the reducing system containing Grx, GRD, GSH, and NADPH by the reduction of a disulfide bond of dimer MST (Figure 6). The result of a preliminary study on the activation mechanism of MST by human Grx1 mixed with the reducing system (Figure 7) also supported the conclusion. Although Grx possesses higher redox potential than does MST or GSH, Grx enzymatically reduces the oxidized form of MST. This is like the case of thioredoxin 1 that can be reduced by Grx with the reducing mixture [52].
